# Rapid Design and Fabrication of Body Conformable Surfaces with Kirigami Cutting and Machine Learning

**DOI:** 10.1002/advs.202522787

**Published:** 2026-02-10

**Authors:** Jyotshna Bali, Jinyang Li, Jie chen, Suyi Li

**Affiliations:** ^1^ Department of Mechanical Engineering Virginia Tech Blacksburg Virginia USA; ^2^ Macromolecules Innovation Institute Virginia Tech Blacksburg Virginia USA; ^3^ VT Made Virginia Tech Blacksburg Virginia USA

**Keywords:** kirigami, surrogate modeling, wearables

## Abstract

By integrating the principles of kirigami cutting and data‐driven modeling, this study aims to develop a personalized, rapid, and low‐cost design and fabrication pipeline for creating body‐conformable surfaces around the knee joint. The process begins with 3D scanning of the anterior knee surface of human subjects, followed by extracting the corresponding skin deformation between two joint angles in terms of longitudinal strain and Poisson's ratio. In parallel, a machine learning model is constructed using extensive simulation data from experimentally calibrated finite element analysis. This model employs Gaussian Process (GP) regression to relate kirigami cut lengths to the resulting longitudinal strain and Poisson's ratio. With an R^2^ score of 0.996, GP regression outperforms other models in predicting kirigami's large deformations. Finally, an inverse design approach based on the Covariance Matrix Adaptation Evolution Strategy (CMA‐ES) is used to generate kirigami patch designs that replicate the in‐plane skin deformation observed from the knee scans. This pipeline was applied to three human subjects, and the resulting kirigami knee patches were fabricated using rapid laser cutting, requiring less than a business day from knee scanning to kirigami patch delivery. The low‐cost, personalized kirigami patches successfully conformed to over 75% of the skin area across all subjects. The kirigami‐inspired, machine‐learning‐driven design and fabrication pipeline presents a balanced trade‐off between conformability performance and cost for personalizing wearables, thus establishing a foundation for a wide range of new functional devices.

## Graphical abstract

By integrating the principles of kirigami cutting and data‐driven modeling, this study aims to develop a personalized, rapid, and low‐cost design and fabrication pipeline for creating body‐conformable surfaces around the knee joint. This pipeline presents a balanced trade‐off between conformability performance and cost for personalizing wearables, thus establishing a foundation for a wide range of new functional devices.

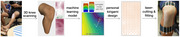

## 1 Introduction

Over the past decades, body‐conformable and wearable devices have attracted wide interest from many communities. These wearable devices aim to mimic the mechanical properties of the underlying skin, enabling them to conform to its non‐uniform deformation and irregular shape. As a result, wearable devices can operate with minimal interference with the dynamic body motions, while ensuring bio‐compatibility, functional performance, comfort, and durability. A large subset of the conformable devices takes the form of a surface with different thicknesses. So matching their in‐place surface deformations with the underlying skin plays a critical role. To this end, researchers have developed two complementary approaches — structural design and material engineering [1, 2]. The structural design approach employs tailored geometric transformation via structure patterning techniques, such as island bridges and network architectures [3] using filamentary serpentine and wavy ribbons [4, 5, 6], fractal‐inspired and hierarchical motifs [7, 8], spirals and springs [9, 10], helical coils [11], honeycomb grids and chains [12, 13], auxetics [14], Origami [15, 16], and Kirigami [17, 18, 19]. The material engineering approach, on the other hand, exploits the constituent material's stretchability and adhesion [20, 21] to improve conformability. This approach employs elastomeric polymers and composites [22, 23, 24], hydrogel polymers [25], liquid metals [26, 27], shape memory polymers [28], and fabrics [29, 30]. These structural design and material engineering techniques can be seamlessly combined with advanced manufacturing and integration technologies [31, 32], such as printing and coating [33, 34], thin film deposition and transfer printing [35], as well as self‐assembly and morphing [36]. Implementing these skin‐conformable surfaces can advance healthcare and biomedical wearables [3, 37, 38, 39], soft robotics and human‐machine interfaces [40], energy harvesting and wearable power systems [15, 41, 42], smart textiles and fashion technologies [30, 39, 43], as well as sports and performance monitoring systems [39, 44].

While surface conformability in wearable devices has significantly enhanced their functionalities, several challenges still limit their widespread applications. Mechanical mismatch is one of the most prominent ones, and it often results in discomfort to the users and significantly reduces the wearable performance [1]. This challenge highlights the importance of incorporating *personalized* and *accurate* skin deformation knowledge into the wearable design [2]. Few state‐of‐the‐art wearable devices are personalized — most of them adopted a non‐ideal “one size fits all” strategy, assuming that in‐plane softness is sufficient for conformability. (That is, one typically assumes that “if the wearables are sufficiently soft, they should always fit the human body.”) Such simplification inevitably leads to performance tradeoffs and mechanical mismatches. On the other hand, designing for individual human variability is an enormous task with a seemingly infinite variable space and complex objectives. As a result, personalized design can be quite time‐consuming and resource‐intensive.

Therefore, in this study, we aim to formulate a design and fabrication pipeline to overcome these challenges and create low‐cost, highly individualized, and body‐conformable wearable surfaces. To this end, we will adopt Kirigami cutting and data‐driven surrogate modeling techniques and focus on the knee surface as the case study. The principle of Kirigami cutting has been widely used to impart flexibility, stretchability, conformal contact, adhesion, and programmable shape change to over‐the‐body applications, such as wearable bio‐electrodes, electronic skins, sensors [18, 45], implantable [46, 47], e‐textile [48], thermo‐regulation devices [49], as well as other multifunctional integrated platforms [17]. Kirigami‐cut surfaces have a unique ability to stretch anisotropically through the opening of their cuts and internal deformation, which can be designed to match the local skin deformation underneath while maintaining contact and breathability [50]. This ability has been utilized to design wearable patches around movable joints, such as the shoulder [51], knee [52, 53], elbow [54], and wrist [55, 56]. However, these Kirigami patches are either manufactured with minimal individualization or lack the rapid design and fabrication potential for scaling up.

**FIGURE 1:**
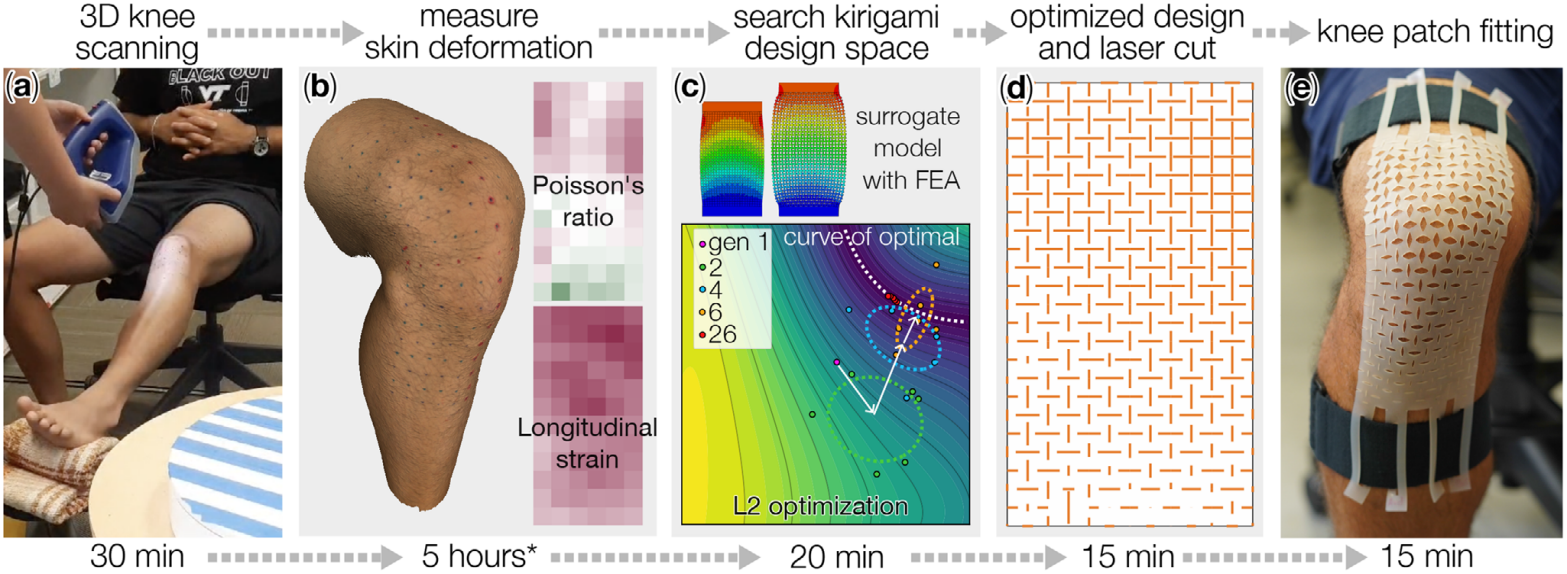
Flowchart of the rapid design and fabrication pipeline of personalized Kirigami knee patch. Note that the second step — measure skin deformation — is completed manually in this study due to the need to manually transfer data between different proprietary software. Developing an integrated and automated software package could further speed up this process. (Video S1).

In this study, we achieved the rapid design and fabrication of conformable knee patches by carefully tuning their cutting parameters (i.e., the length of every cut) to accurately match the complex 3D skin deformation. More specifically, we first gathered the skin deformation data by 3D scanning the subject's knee surface at the initial and final knee joint angles (Figure 1a). These scanning data allowed us to quickly extract skin deformation information, including longitudinal strain and Poisson's ratio (heatmaps in Figure 1b). Meanwhile, we conducted Finite Element Analysis (FEA) and physical testing of silicone rubber Kirigami sheets with a uniform cut pattern to correlate the cut parameters to their elastic deformation. These experiment‐validated FEA data enabled us to construct an accurate, high‐resolution surrogate model for inverse design (Figure 1c). We compared multiple machine learning models, Gaussian Process regression (GP, also known as Krigin) [57, 58, 59], Polynomial regression (PR), Gradient Boosting Regression (GBR), and Random Forest Regression (RF), among which GP is the most accurate for the Kirigami design problem. Finally, we adopted a state‐of‐the‐art optimization algorithm, Covariance Matrix Adaptation Evolution Strategy (CMA‐ES) [60] to search for the optimal Kirigami cut designs that conform to skin deformation. The optimized design can be quickly manufactured using a laser cutter (Figure 1d,e).

Thanks to the design flexibility and simple fabrication of Kirigami, the design and fabrication pipeline can be (i) easily personalized because only a simple 3D scanning is required, (ii) with a low cost because laser cutting is widely accessible, and (iii) rapid so that it takes only a working day (about 6.5 h) between knee scanning and the Kirigami patch completion.

The rest of the paper details the different steps of the Kirigami knee patch design and fabrication pipeline, discusses the outcome, and outlines its future applicability.

## 2 Methodology

### 2.1 Obtaining The Design Target: User's Knee Skin Deformation

**FIGURE 2:**
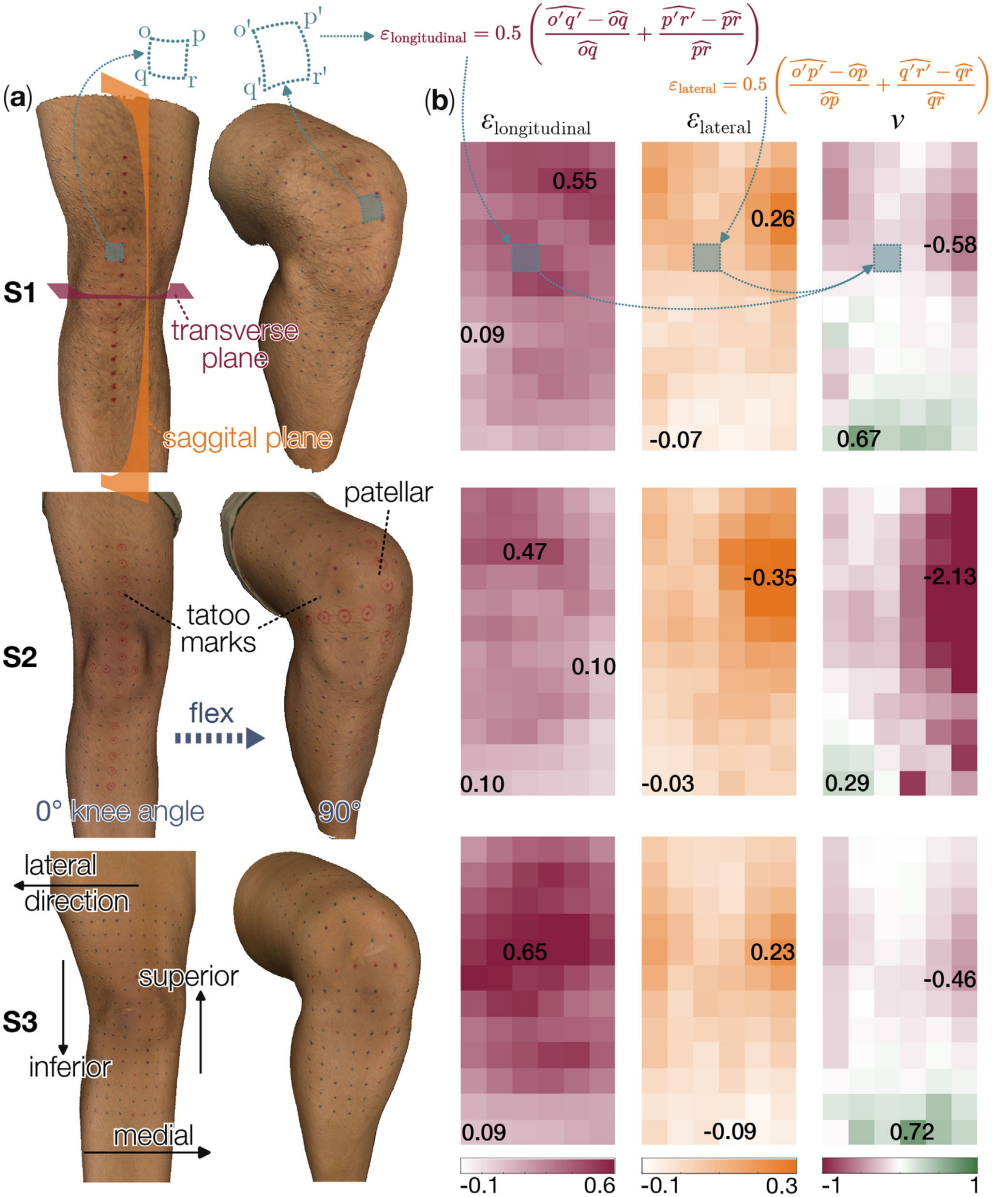
Scanning and measuring the subjects' knee surface deformation. (a) Reconstructed knee surfaces of the 3 subjects (i.e., S1, S2, and S3), using the 3D scanning data. (b) Heatmaps showing the longitudinal, lateral strain, and Poisson's ratio distribution from the 0° to 90° knee flex. The unit cells with the maximum and minimum values in each heatmap are labeled, further highlighting the variety between different subjects. Note that the length variables (e.g., $\hat{op}$ and $\hat{q^{\prime }r^{\prime }}$) in the strain calculations are geodesic distances on the 3D knee surface.

To conduct this human participant research, we first obtained approval from the Virginia Tech Institutional Review Board (IRB No. 24‐775) and written consent from all the volunteers. Then, to measure the skin deformation in an accurate, reliable, and consistent setup, we used a temporary tattoo marker to draw a grid directly on three volunteer subjects' right knee skin (Figure 2a). The grid has a size of 240 × 180mm with a square unit cell size of 15 mm, totaling 16 rows and 12 columns. It spans from the medial to the lateral epicondyles and extends uniformly from the superior to the inferior region of the patella. The skin deformation pattern was then captured with a high‐resolution, handheld 3D Scanner (Artec Eva), as the subjects flexed their knee joints from a relaxed 0° to the 90° angle. The scanner generated a dense point cloud, which was then converted into a smoothed polygon model and exported in the OBJ format. Then, we post‐processed the data in the open‐source GigaMesh software to measure the geodesic distances between adjacent marker points [61]. By comparing these geodesic distances before and after knee flexure, we can calculate the averaged longitudinal strain, lateral strain, and Poisson's ratio of each cell. Overall, this process allowed us to map the 3D surface scans of the knee skin into a flattened 2D strain and Poisson's ratio heatmap (Figure 2b).

The longitudinal strain heatmap of all three subjects showed positive stretches in almost all regions of the knee, which agrees with the established literature on skin deformation of the lower body [62, 63]. Such longitudinal stretch is particularly prominent around the patellar region, as indicated by a darker maroon color in the first column of heatmaps in Figure 2b. In comparison, the magnitude of lateral strain appeared roughly halved, and a greater lateral stretch, as denoted by the orange color, was observed on both medial and lateral sides of the patella in all three subjects. Notably, the regions below the patella exhibited small lateral compression, which concentrated on the most inferior portion of the scanned region. Correspondingly, the Poisson's ratio heatmap shows predominantly negative values (third column of heatmaps in Figure 2b), indicating that the knee skins are expanding in their surface area during knee joint flexing. Meanwhile, a small region of positive Poisson's ratio — corresponding to lateral compression and longitudinal expansion — occurs along the inferior portion of the knee scan (green regions in this heatmap). In what follows, the longitudinal strain and Poisson's ratio heatmaps will serve as the objective functions for the Kirigami design.

### 2.2 Constructing The Kirigami's Design Space

**FIGURE 3:**
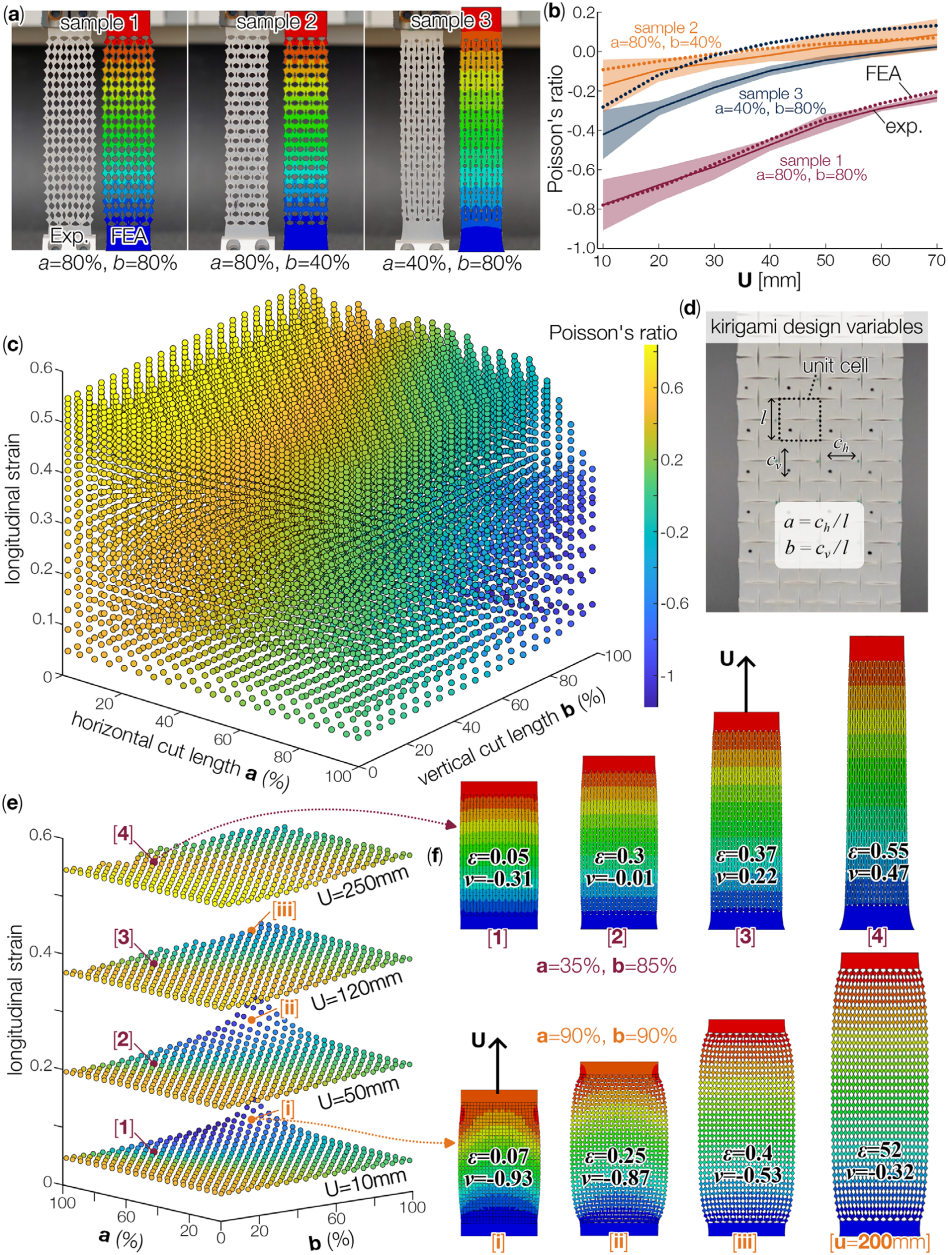
Populating the design space of Kirigami with experimentally‐calibrated finite element analysis (FEA). (a) Comparing the Kirigami sheets' deformation pattern between FEA and experiment (with top end displacement U=70mm). (b) Comparing the Poisson's ratio between FEA and experiment. Here, the solid lines are averaged test data, and the shaded bands are standard deviations from the readings of 12 unit cells in the middle of the samples. The dashed lines are FEA predictions. (c) Populated design space, where each marker corresponds to a unique combination of Kirigami cut design and longitudinal strain, and the color of the marker represents the corresponding Poisson's ratio. (d) A close‐up view of the Kirigami design variables. (e) A subsection of this design space, showing a few slices corresponding to U=10, 50, 120, 250mm. (f) Detailed FEA simulation outcomes, where [i‐iv] and [1‐4] highlight two Kirigami designs.

The skin deformation heatmaps from the three subjects showed a wide range of longitudinal strain and Poisson's ratio combinations. Therefore, in this section, we explore whether the Kirigami sheet can capture such variation with intentional design. To this end, we adopted the classical Kirigami design with a tessellated “cross cut” pattern (Figure 3). We chose this design for two reasons. First, it can achieve a large range of positive and negative Poisson's ratios at different strain levels. Second, the square cut pattern is relatively simple to design. This second reason is particularly crucial for this study because its goal is to explore how to design and fabricate a personalized conformable surface *rapidly* (with low computational and manufacturing costs).

The cross‐cut kirigami pattern can be divided into square‐shaped unit cells, which conveniently correspond to the grid pattern used in the 3D knee surface scanning. For each unit cell, the lengths of its horizontal and vertical cuts are denoted as *a* and *b*, respectively. They are percentage values indicating the cut length with respect to the unit cell size (Figure 3d). For example, the three Kirigami samples shown in Figure 3a were made from a 140 × 40 × 2 mm silicone rubber sheet (Dragon Skin 20), consisting of an array of square unit cells with 10 × 10 mm in size. The first sample had a uniform cut pattern of *a* = 80% and *b* = 80%, meaning the lengths of horizontal and vertical cuts are both 80% of the unit cell size. In contrast, the second and third samples had unequal horizontal and vertical cut lengths. We then clamped the Kirigami sheets at both ends and uniformly stretched them on a universal tensile tester machine (Instron 6800 Series with a 100N load cell). To measure the longitudinal strain and Poisson's ratio with minimal boundary effects, we focused on the cells in the middle and captured their deformed shapes with a high‐resolution camera (Sony α7C II). These images can be analyzed with the image processing toolbox in MATLAB to obtain the deformation curves shown in Figure 3b.

In parallel to the experiments, we also conducted finite element analysis (FEA) of the same Kirigami samples using 2D shell elements (ABAQUS, CPS4R) and Neo–Hookean constitutive properties (shear modulus, μ = 0.207*MPa* [64]). In these FEA models, the Kirigami cuts were represented by the “seam” feature, which partitions the sheet horizontally and vertically to allow structured meshing (seed size 0.5). Similar to the experiment setup, we applied longitudinal stretching displacement at the top boundary, while fully fixing the bottom boundary. The longitudinal strain and Poisson's ratio of the unit cells in the middle region of this simulated Kirigami sheet were then compared with the experimental data, which showed a good agreement (Figure 3b). Therefore, the finite element model can reliably capture the Kirigami sheet's deformation characteristics, so we could use it to investigate different Kirigami cross‐cut patterns and explore the full design space.

To this end, we conducted extensive simulations to populate Kirigami's design space. We modeled and simulated 441 Kirigami sheets that have the same overall size but different cut lengths (Figure 3c–e). These Kirigami sheets were 220 × 120mm, consisting of 18 rows and 12 columns of 10 × 10mm unit cells. Within each Kirigami sheet, the cut lengths are uniform, but between the different Kirigami sheets, the horizontal and vertical cut lengths vary independently from 5% to 95% of the unit cell size, with an increment of 5%. In addition, 1% and 99% cut lengths were included to define the minimum and maximum cut limits of the design space. We then simulated these Kirigami sheets' longitudinal deformation under 10 to 250 mm of stretching displacements, with increments of 10 mm. In every simulation, the deformations of the middle four unit cells were used to calculate the output longitudinal strain and Poisson's ratio. Therefore, the output of these 441 simulations populate the full design space of Kirigami, as shown in Figure 3c.

This full design space plot confirmed that, with a tailored horizontal and vertical cut length and appropriate stretching, the Kirigami sheet can achieve a wide range of longitudinal strain and Poisson's ratio combinations. Here, each circular marker represents a Kirigami sheet of a particular horizontal and vertical cut length, pulled by prescribed stretching displacement. As a Kirigami sheet is being stretched, its longitudinal strain increases at a greater rate at first, and then slows down later after most of the cuts have opened up. Interestingly, Poisson's ratio increases monotonically as its longitudinal strain increases, which is reflected by the color distribution of the circular markers. Kirigami sheets of smaller horizontal and vertical cuts can exhibit a positive and increasing Poisson's ratio throughout their deformation range. On the other hand, Kirigami sheets with longer cut lengths could show a transformation from being auxetic to non‐auxetic as their stretch increases. Such a trend can be more visible by slicing and highlighting several layers of the 3D Design space (e.g., 10, 50, 120, and 250 mm deformation level as shown in Figure 3e.

To better illustrate the Kirigami's deformation characteristics, Figure 3f details the simulation outcomes of two samples — one has a moderate cut length *a* = 35%, *b* = 85%, and the other has a longer cut at *a* = 90%, *b* = 90%. As the former Kirigami is stretched from 0.05 to 0.55 strain levels, its rectangular “facets” would rotate, thus expanding the lateral dimensions and creating the classical auxetic behavior. In other words, the kinematics of facet rotation dominate the deformation behavior at low stretching strain. However, as this Kirigami's stretch increases toward the 0.55 strain level, the facet rotation reaches its kinematic limits. As a result, the facets stop rotating, but become elastically deformed, exhibiting the positive Poisson's ratio. Importantly, longer Kirigami cuts increase the kinematic limit of facet rotation. For example, the other Kirigami sheet with a 90% cut is stretched significantly by 200 mm in our simulation. Its Poisson's ratio increases from –0.93 to –0.32; however, it did not reach the kinematic facet rotation limit.

In summary, with the help of extensive FEA simulations of Kirigami sheets with varying horizontal and vertical cut lengths, we were able to fully populate their design space. This space will serve as the foundation for conformable surface design in the following sections.

### 2.3 Surrogate Modeling of The Kirigami Structure–Property Relationship

While the populated design space from the finite element model was comprehensive, it was still sampled by discrete cut lengths with 5% increments. Such a non‐continuous nature can present limitations for inverse designing the Kirigami cut based on knee skin deformation. For example, precisely matching the knee skin deformation might require cut lengths not sampled by the finite element simulations. Moreover, multiple Kirigami cut lengths could give similar deformation characteristics to match the knee skin deformation (i.e., many‐to‐one mapping). One can certainly address this issue by running more FEA simulations to increase the resolution of the design space, but the computational cost can be prohibitive. Therefore, we built a surrogate model using machine learning methods to accurately search Kirigami cut sizes with the least deviation from the design objectives.

#### 2.3.1 Variables Shifting for Surrogate Modeling

**FIGURE 4:**
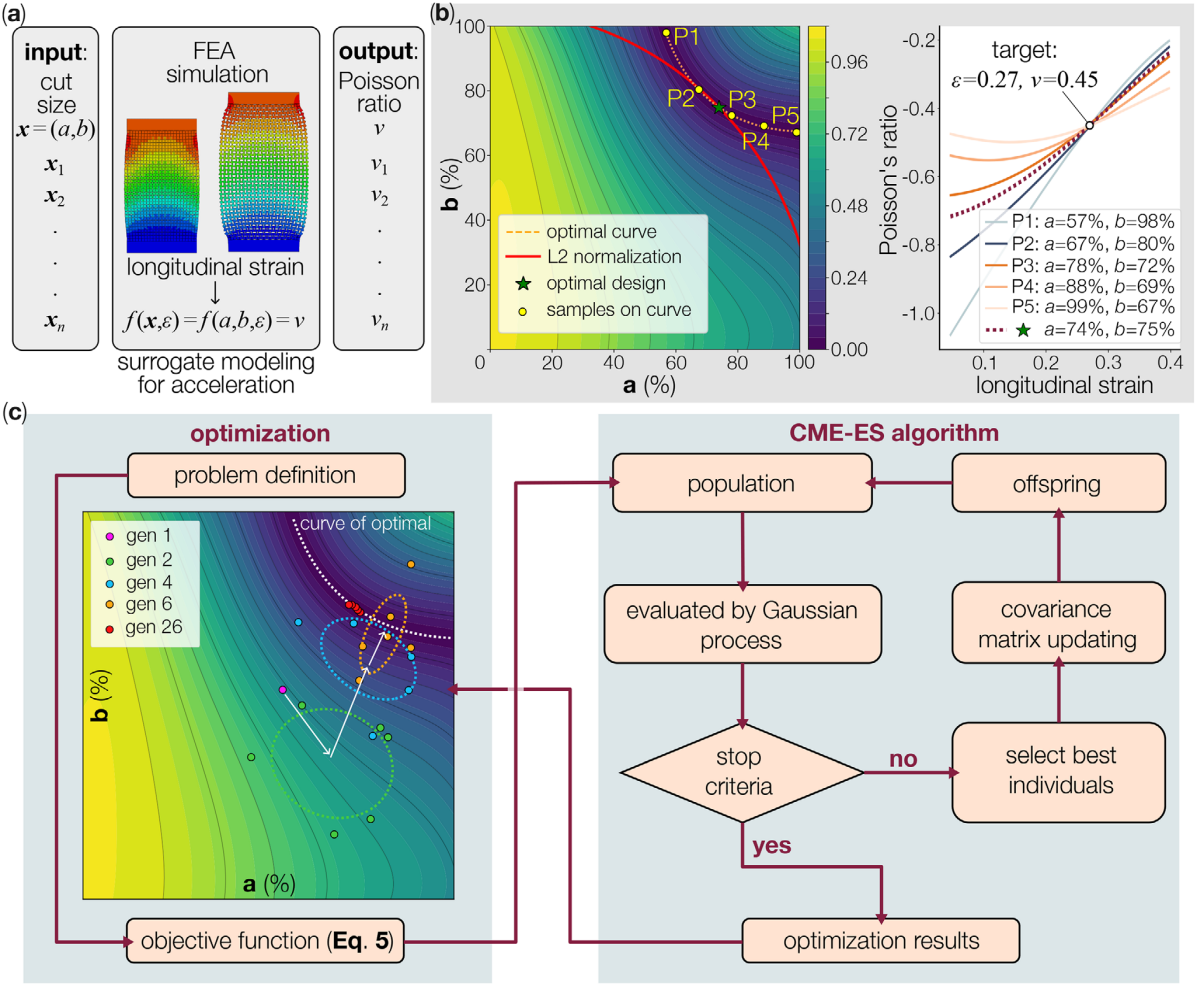
Elements of the inverse design methodology. (a) The input and output architecture of the surrogate modeling. (b) L2‐Normalization for design candidate selection. In the design space plot on the left, we selected 6 design candidates on the optimal curve (i.e., P1‐P5 and the optimal candidate). Then their longitudinal strain vs. Poisson's ratio responses are plotted on the right. (c) The flow chart of the optimal Kirigami design: The initial data was sampled randomly at first, as shown by generation 1. After a few iterations, it converges to our optimal solution at the 26th iteration.

We attempted to use the finite element simulation outcome to generate the surrogate model. However, there is no one‐to‐one mapping from the geometric design variables, i.e., *a* and *b*, to the targeted deformation characteristics, i.e., longitudinal strain and Poisson's ratios. Considering the longitudinal strain increases monotonously in the tensile simulation, we decided to use it as another input to the surrogate model in addition to the two cut length variables. As a result, the surrogate model will output the Poisson's Ratio as the prediction (Figure 4a).

To ensure accuracy, we compared the following regression models: Gaussian Process Regression (GP, also known as Kriging), Random Forest Regression (RF), Polynomial Regression (PR), and Gradient Boosting Regression (GBR). As shown in Table 1, the GP provides the highest accuracy. Our surrogate modeling can be represented as follows:

(1) $$\mu =FEM\left(a_{x},b_{y},\varepsilon \right)\approx f\left(a_{x},b_{y},\varepsilon \right)$$

where *f* represents the surrogate models, we use the cross‐validation method to test its accuracy in 10 folds using the 441 samples. In 10‐fold cross‐validation, the dataset is split into 10 equal parts. Then, we process the modeling 10 times, with each part being the test set once. Finally, the modeling accuracy is evaluated as the mean of the 10 models. The model accuracy is shown in Table 1. Clearly, the Gaussian Process Regression presents the most accurate prediction (*R* ^2^ = 0.997), so we use it as the basis of the surrogate model hereafter. For readability, we provide a brief introduction to GP. Additional details are available in Ref. [65].

(2) $$\mu \left(x^{*}\right)=GP\left(x^{*}\right)=GP\left(a_{x},b_{y},\varepsilon \right)=k^{⊤}{\left(K+\sigma_{n}^{2}I\right)}^{-1}y$$

where μ(x*) is the mean value of the response of the input data point x*. In Equation (2), **k** ^⊤^ and **K** are calculated from the Gaussian kernel function. **K** is a matrix, and its elements can be represented by

(3) $$K_{ij}=k\left(x_{i},x_{j}\right)=\sigma_{n}^{2}\exp\left(-\;\frac{∥x_{i}-x_{j}{∥}_{2}^{2}}{2l^{2}}\right)$$

with some hyper parameters σ, *l* where *x*_*i*_, *x*_*j*_ are the input data sampled from the training dataset and $y={\left[\begin{array}{cccc} y_{1} & y_{2} & \cdots & y_{n} \end{array}\right]}^{⊤}$ are the responses to the sampled data. For **k** ^⊤^, it calculates the distance to all sampled data points,

(4) $$k^{⊤}={\left[\begin{array}{cccc} k(x^{*},x_{1}) & k(x^{*},x_{2}) & \cdots & k(x^{*},x_{n}) \end{array}\right]}^{⊤}$$

**TABLE 1:** Performance comparison of surrogate models.

**Surrogate model**	**R^2^**
Gaussian Process Regression	0.9969
Gradient Boosting Regression	0.7961
Random Forest Regression	0.5120
Polynomial Regression (degree=3)	0.9124

### 2.4 Inverse Design and Fabrication of The Kirigami Knee Patches

With the accurate surrogate model, we can perform inverse design using optimization techniques. Although we simulated extreme values of design variables (i.e., *a* = 99%, and *b* = 99%), it is nearly impractical to fabricate them. As a result, the maximum horizontal and vertical cut lengths are limited to 90%. More importantly, our surrogate model reveals that, for a given combination of longitudinal strain ε and Poisson's ratio ν, there are an infinite number of cut length solutions. To identify a unique and optimal design, we introduce the L2‐normalization — a technique in machine learning to overcome overfitting. For example, Figure 4b shows a curve of optimal solutions, where all the Kirigami cut designs on this curve can minimize our objective function: ε = 0.27, ν = 0.45. However, some of these points are at the boundary of the design space with a large horizontal cut and a small vertical cut, or vice versa. This may cause excessive stress concentration. To solve this problem, L2‐normalization selects the solution that is closest to the origin of the design space to maintain relatively equal cut lengths in both directions. In Figure 4b, we selected 6 design candidates on the optimal curve, including the L2‐normalized design, and plotted their longitudinal strain vs. Poisson's ratio responses. All these designs can satisfy our design objective; however, the L2‐normalization selects the design closest to the origin, giving the most even cut lengths. The L2‐normalization can help minimize the heterogeneity by ensuring the horizontal and vertical cut lengths are as uniform as possible.

After establishing the surrogate model and design optimization procedures, we proceed to inverse design of the Kirigami cut according to the 3D scanned skin deformation. That is, the 3D knee scanning data produced a set of heat maps containing 12 × 6 (row × column) grid cells, each has a unique combination of longitudinal strain and Poisson's ratio (Figure 2). Correspondingly, the Kirigami knee patch should also have 12 × 6 unit cells. The cut lengths of each cell should be selected to match the corresponding knee scan data using the aforementioned surrogate model and design optimization procedures (Figure 4).

**FIGURE 5:**
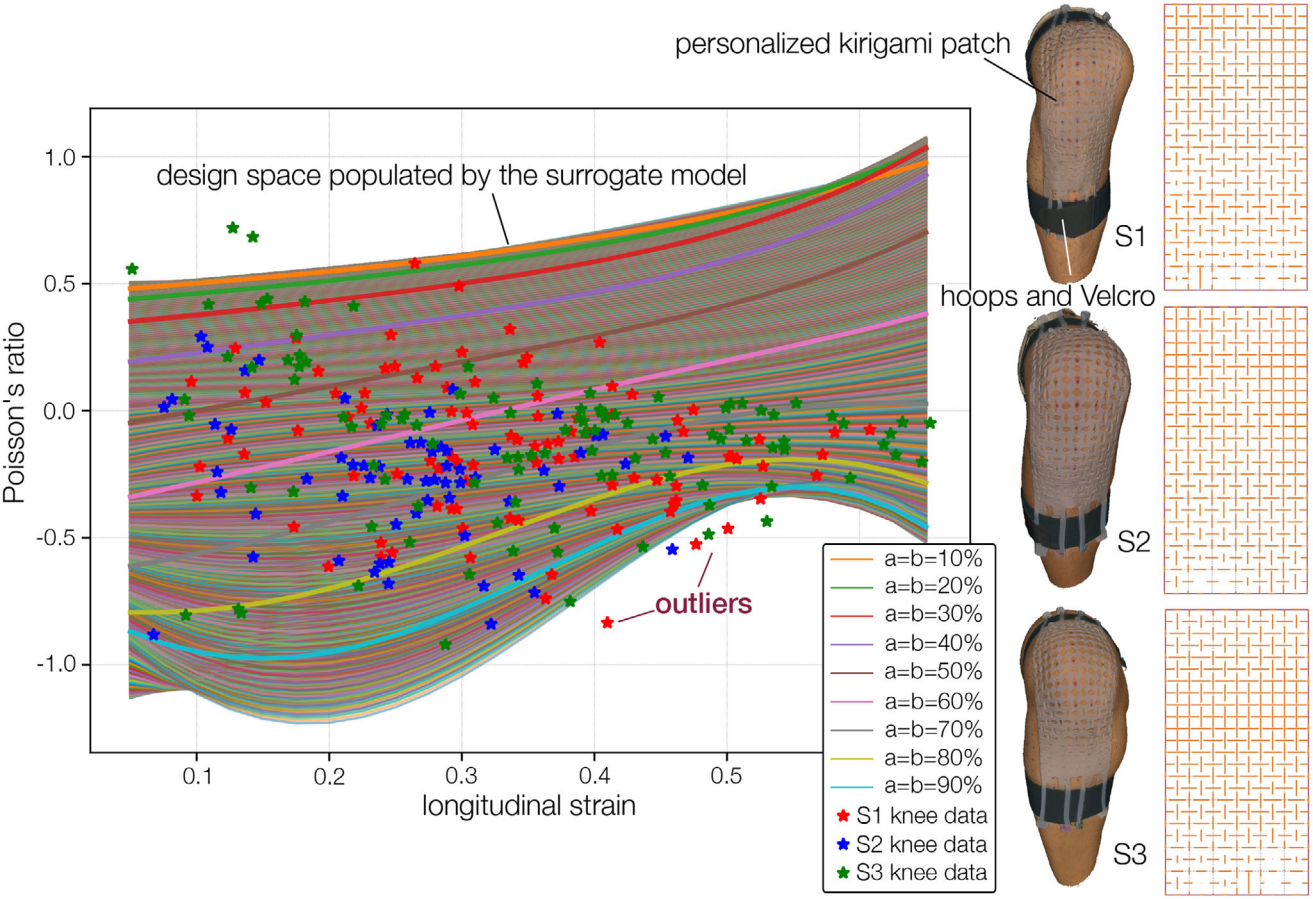
The final, personalized Kirigami patches for the three subjects. (left) In this plot, each star‐shaped marker represents the longitudinal strain–Poisson's ratio combination of a grid cell on the subject's knee surface. They are the same data from the heatmaps in Figure 2b, but plotted differently. Meanwhile, each thin curve in this plot is the longitudinal strain–Poisson's ratio response corresponding to a unique Kirigami cut design. These curves are generated by the surrogate model, which is based on the FEA simulation data in Figure 3c. Essentially, the optimization procedure laid out in Figure 4c finds the curve that best matches each star‐shaped marker. (right) The final Kirigami patch designs. The images in the left column are the reconstructed 3D scanning data from the subjects wearing their Kirigami patches, and the images in the right column are the corresponding Kirigami designs.

To this end, we first compared the target unit cell deformations from the knee scan data and the achievable unit cell deformation from the Kirigami surrogate model. By plotting these two sets of data together (Figure 5, left), we found that the large design space of Kirigami could indeed cover the variability of subjects' skin deformations. While outliers exist (aka, there are several unit cells on subjects' knees that show deformations beyond Kirigami's design space), they do not venture far away from Kirigami's capability. Therefore, we could formulate the personalized Kirigami design optimization as:

(5) $$\begin{array}{cc} \min_{a,b}:\; & l={\left(\mu_{\text{scan}}-GP\left(a,b,\varepsilon_{\text{scan}}\right)\right)}^{2}+\lambda \left(a^{2}+b^{2}\right)\\ \text{s.t.:}\; & 0\leq a\leq 90\%,\;0\leq b\leq 90\% \end{array}$$

where *l* is the objective function measuring the difference between the targeted Poisson's ratio from the 3D knee scan data and the predicted Poisson's ratio from the surrogate model, and λ is the penalty coefficient of the L2 normalization. The above optimization process is used to design the cut lengths of all unit cells in the Kirigami patch. The optimization problem was solved using the state‐of‐the‐art optimization CMA‐ES, which encodes the input variables and forms a population to initiate the search process. After selection, crossover, and mutation operators that generate the offspring population to optimize the objective function, the process terminates upon reaching the specified stopping criteria, yielding the current optimal solution (Figure 4c).

Since the knee surface's longitudinal strain and Poisson's ratio can differ significantly between adjacent grid cells, the corresponding Kirigami cut length also differs between adjacent unit cells. Therefore, for the cuts at the boundary between adjacent cells, we performed a simple averaging and skewing. The final design was exported as a vector image file for laser cutting. To fabricate the Kirigami knee patch, we first prepared a 2mm thin silicone rubber sheet with the help of a film applicator (DragonSkin 20). After curing, the rubber sheet is cut with a CO_2_ laser cutter (Trotec Speedy 360, 40% of max power, 0.4% of max laser head speed, and 1000 Hz excitation frequency). Note that several additional hoops were designed at the two ends of the Kirigami patch so that we can use Velcro straps to quickly attach the Kirigami patch to the subjects' knees (Figure 5, right).

## 3 Results and Discussion

### 3.1 Overall Conformability Performance

**FIGURE 6:**
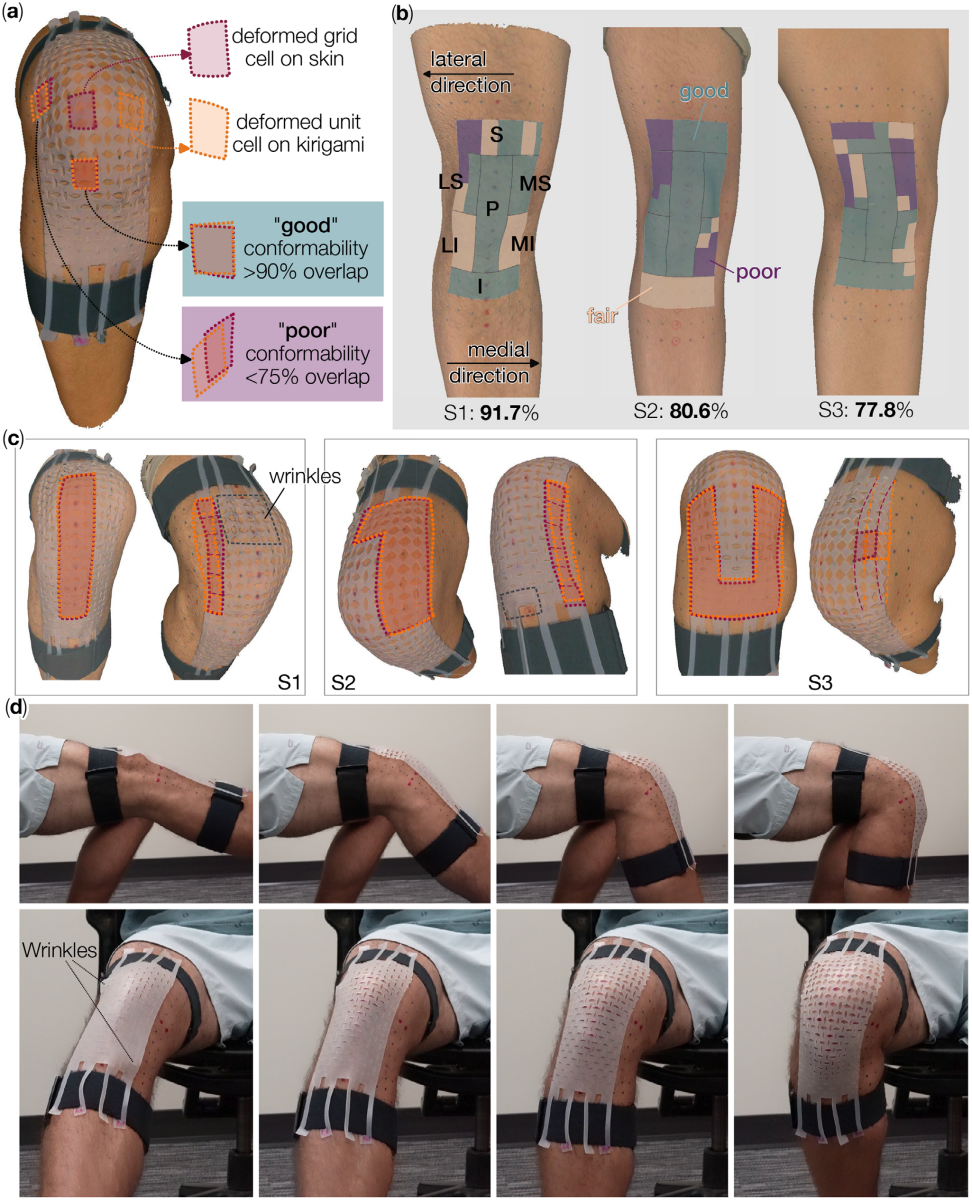
Conformability analysis of the three patches. (a) 3D scanning image of subject S1 wearing its personalized Kirigami patch. Here, several grid cells on the knee surface and unit cells in the Kirigami are highlighted to define conformability. (b) A summary of conformability scores for the three Kirigami patches. (c) Close‐up views highlighting a few good and poor conformability regions. (d) A close‐up look at the subject S1's kirigami patch conformability at different knee bending angles (Video S2).

Here, we quantify “conformability” based on the areal overlap between the unit cells of the Kirigami patch over their corresponding grid cells on the subject's knee surface, when the knee is flexed at 90 degrees. A visual representation can be seen in Figure 6a, where dashed maroon lines define a grid cell drawn on the skin, and dashed orange lines define the corresponding unit cell in the deformed Kirigami patch. When the overlap between the two cells is equal to or more than 90% of their surface area, we considered the conformability “good.” Meanwhile, an areal overlap greater than 75% and less than 90% indicates a “fair” conformability; and below 75% overlap is considered “poor.” Figure 6 summarizes different regions of conformability on the three subjects.

To assist in analyzing the conformability, we first divided the patch into 7 regions with respect to the patellar (Figure 6b), including

- **S**: region Superior to the patella (top 2 rows of unit cells);
- **I**: region Inferior to the patella (bottom 2 rows);
- **LS**: region on the Lateral side of the leg, and Superior to the patella;
- **MS**: region on the Medial side, and Superior to the patella;
- **LI**: region on the Lateral side, and Inferior to the patella;
- **MI**: region on the Medial side, and Inferior to the patella;
- **P** Patellar region, which is enclosed by the S, LS, MS, LI, MI, and I regions.

With the help of these seven regions, one can more easily analyze the individual Kirigami patches and their trend of conformability performance. For example, subject S1's Kirigami patch showed some out‐of‐plane wrinkling in the center of region S, likely due to the concentrated stretching from the Velcro straps and fitting loops (dashed grey lines in Figure 6c). Regardless, the overall conformability is still satisfactory among these unit cells. Some small out‐of‐plane wrinkling was also visible at the bottom of region I, but it did not significantly hinder this region's overall conformability either. Overall, the unit cells on the center‐right portions of the Kirigami patch, including the middle half of region S, MS, and the whole region of P and I, showed good conformability with more than 90% areal overlap. However, the conformability performance can start to drop toward the medial and lateral sides. For example, the outer region of LS showed an areal overlap decreasing below 75%, as well as the lateral edge of region S. The LI and MI regions showed a fair conformability score. Regardless, the overall conformability of the Kirigami knee patch designed for subject S1, as summarized in Figure 6b, still showed a promising outcome. Out of the 72 unit cells in total, 66 cells achieved at least 75% areal overlap between the knee surface and the patch (i.e., fair and good conformability), giving a 91.67% satisfactory ratio.

Despite the large variation between the three subjects regarding their gender, physical activity level, and age, the performance of their Kirigami patches followed a similar trend: Good conformability in the center with a degraded performance near the lateral and medial edges. For subject S2, 58 cells out of 72 unit cells achieved a fair or good conformability score (an 80.56% satisfactory rate), and for subject S3, 56 unit cells conformed satisfactorily (or 77.78% score). These results indicate that our proposed design pipeline can accurately accommodate human body variability.

In addition, it is worth noting that, even though the cut geometry is designed to fit knee skin deformation at 90° joint angle, the kirigami patch can still conform well at the smaller angles (Figure 6d) (except for the additional wrinkling appearing near 0° joint angle, which is the subject of the following subsection). To this end, we further examined another set of 3D scan data from subject S1's left knee at 0°, 45°, and 90° joint angles (Appendix Section A). Using these data, we can calculate the knee deformation from 0 to 45 and from 0 to 90 degrees, respectively. Comparing these two sets of data reveals that the knee skin's strain increases monotonically as the joint bends further, but the Poisson ratios remain in a similar range. Therefore, the flexible kirigami patch can accommodate the complexity of knee skin deformations and conform to smaller joint angles.

### 3.2 Critical Discussions on the Local Conformability Mismatches

Despite the overall satisfactory performance, deformation mismatches between the knee skin and kirigami still emerged in the low‐conformability regions (e.g., the lateral edge of region S and the upper half of region LS, as shown in Figure 6c). Such discrepancies originate from three underlying simplifications that we accepted for the purpose of enabling rapid personalization pipelines without excessively high cost in computation and fabrication. Here, we detail these simplifications and their potential impacts on conformability.

#### 3.2.1 Knee Skin's Initial Curvatures Before Flexion

In this study, we design kirigami cut geometry to fit knee skin deformation only at the 90° joint angle. This setup intrinsically assumes that the kirigami, when flat and undeformed, can directly conform to the knee skin before flexion (i.e., at 0° joint angle). Such an assumption obviously neglected the complex knee surface curvatures at the beginning, especially around the patella. From Figure 6d, it is clear that when the knee is straight, local wrinkles emerge near the patella and near the lower fitting strap, indicating a mismatch. However, overall, there are no significant gaps between the kirigami patch and the knee surface. As the subject starts to flex his knee, the local wrinkling quickly disappears. Therefore, the omission of the knee skin's initial curvatures at 0° joint angle negatively impacted the initial comformability, but the corresponding wrinkling is local and relatively small in magnitude.

#### 3.2.2 Uniform Kirigami Designs in the Surrogate Model

In finite element simulation and surrogate modeling, the kirigami sheets have a homogeneous design and uniform cut lengths. However, in the final kirigami patch, the cut patterns are heterogeneous. This is another compromise we accepted to allow for rapid design. The elastic constraints between kirigami unit cells with different cut lengths are rather complex, and the design space of the whole kirigami sheet is infinitely large. Considering these factors rigorously in the design pipeline would require iterative methods such as topology optimization, which is time‐consuming and defeats the purpose of rapid personalization.

To minimize the negative impacts from this simplification in our database, we included the following two critical steps in our design pipelines. One is the L2 Normalization detailed in Section 2.4. Multiple kirigami designs can match each grid cell's deformation, but some unit cell design candidates are more heterogeneous than others. Therefore, the L2 normalization can help minimize the heterogeneity by ensuring the horizontal and vertical cut lengths are as uniform as possible. The other critical step concerns the transition cuts between unit cells. In the kirigami patch, there are cuts at the boundary between two adjacent unit cells. If these two cells differ in design, we average and skew these boundary cuts to provide a smoother transition (Appendix Section C).

#### 3.2.3 Shearing Deformation

The cross‐cut kirigami pattern in this study is not designed to accommodate shearing deformation. To assess the potential impacts of this simplification, we used the 3D knee scan data to calculate the internal angle changes of each grid cell from 0 to 90° of knee bending. Such angle changes are quantitative representations of shearing — bigger changes in the grid cell's internal angles mean more significant shearing. The results indicate that shearing deformation exists throughout the upper knee surface (Appendix Section B). However, it is relatively minor. Most of the grid cells have <15° change in the internal angles, except for a few at the front lateral or medial edges. Therefore, the knee skin's shearing deformation is another reason behind the localized mismatch in the final kirigami patch.

In summary, these three assumptions and simplifications are the primary reasons behind the local mismatch between the kirigami patches and the subjects' knee skin. However, it is worth emphasizing that this study does not aim for the best personal conformability; instead, its aim is for reasonably good conformability without excessively high costs. In this regard, this study showed that the rapidly designed and fabricated kirigami patch can still conform reasonably well despite these trade‐offs.

### 3.3 An Application Case Study: Protective Knee Cap

The conformability of the Kirigami patches allows them to deform without hindering the dynamic knee joint deformations. As a result, they can accommodate the skin deformation without peeling off or causing irritation. Moreover, the Kirigami cuts also improved the breathability of the patch by allowing perspiration to escape through the cuts. Therefore, the Kirigami patch can be useful in many applications, such as bandages with increased adhesion (especially near moving joints), protective surfaces, or the basis for wearable electronics.

**FIGURE 7:**
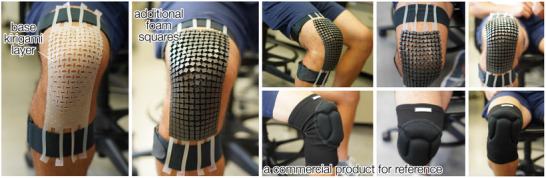
An application demonstration, where impact‐absorbing foams are added to the silicone Kirigami to create a personalized and protective knee cap.

To demonstrate the practical use of conformable Kirigami knee patches, we incorporated an impact‐resistant protective layer onto the silicone Kirigami and created a protective knee cap (Figure 7). For this purpose, we first took a 5 mm, impact‐resistant foam of the same size as the knee patch (Poron XRD, commonly used in sports protective gears) and cut it into squares of half the Kirigami unit cell size (i.e., 7.5 mm, with Trotec Speedy 360 single point laser cutter). Notice the foam was cut completely to avoid introducing additional stiffness to the Kirigami knee patch. Then the foam sheet was carefully aligned with the unit cells of the silicone Kirigami and glued together with silicone adhesive Sil–Poxy. From the side view, top view, and front views in Figure 7, we can see that the protective Kirigami patch conforms well to the knee's flexion with minimal resistance. To compare with the state‐of‐the‐art, the subject also wore a commercially available patch on the same knee (Video S2). The commercial product only uses a few segmented foam patches on the knee surface to provide impact protection, so its conformability is not personalized. This would inevitably introduce additional resistance to the body motion. In contrast, the proposed protective kirigami foam patch offers a mechanically gentler alternative, with enhanced flexibility that closely follows the skin deformation. (Note that this commercial product and our kirigami demonstration are intended only for knee impact protection, not for knee joint stabilization.)

## 4 Summary And Conclusion

In this work, we successfully established a design and fabrication pipeline to develop a personalized body‐conformable surface around the knee joint in ∼6.5 h. The process began by capturing in‐plane skin deformation — from 0° to 90 ° knee flex — by measuring the longitudinal strain and Poisson's ratio of the marker grid drawn on the subjects' anterior knee skin. Simultaneously, we used an experimentally validated FEA model of a silicone‐based Kirigami sheet to train a supervised machine learning surrogate model. This training involved data from 441 FEA simulations of hyperelastic, uniformly‐cut Kirigami sheets, each with a unique combination of horizontal and vertical cut lengths spanning from 1% to 99% of the unit cell size. Then, the Gaussian process regression algorithm used these data to build a correlation between Kirigami cut lengths and the resulting longitudinal strain and Poisson's ratio accurately (*R* ^2^ = 0.996). Finally, an inverse design pipeline using an optimization algorithm of Covariance Matrix Adaptation Evolution Strategy (CMA‐ES) and L2‐normalization employed this surrogate model to generate Kirigami cut patterns that match the knee skin deformation. Implemented with parallel computing, the algorithm achieves a 92% reduction in runtime. This personalized, conformable Kirigami knee patch can be laser‐cut quickly from silicone rubber sheets. We tested the fidelity of this design and fabrication pipeline on three human subjects. The Kirigami knee patches achieved areal overlapping conformability of 91.7%, 80.6%, and 77.8%, respectively. The practical application of the Kirigami knee patch was demonstrated by integrating the personalized Kirigami sheet with a layer of impact‐resistant foam, creating a conformable and protective knee cap.

Thus, our formulated design pipeline successfully captures the complex 3D skin deformation and mitigates mechanical mismatch through a personalized, simple, low‐cost, conformal skin interface. The total duration of our design and fabrication process could be further reduced by using more integrated software packages that can automatically track the grid markers on 3D knee scan data and compute their relative geodesic distances. It's worth emphasizing that this study involves several ingredients that have never been systematically integrated before: (1) a careful measurement of skin deformation, (2) a non‐uniform kirigami pattern aimed to match this skin deformation directly, and (3) a machine‐learning method to enable rapid design. Although these three ingredients have been studied individually before, integrating them into a rapid personalization pipeline for wearables presents a new advancement. Critically, the proposed design and fabrication pipeline, despite the underlying simplifications and assumptions, presents a balanced trade‐off between conformability performance and cost. On the other hand, to extend the kirigami principle to other body parts, one needs to carefully examine the potential impacts from initial skin curvatures and shearing deformation. In conclusion, our proposed process enables rapid, low‐cost design and fabrication of personalized and conformable surfaces. They can serve as the foundation for transdermal patches, electronic skin, and wearable devices, with potential application in biosuits [66].

## Author Contributions

J.B., and J.L. contributed equally to this work. S.L. and J.C. conceived and designed this study. J.B. conducted the experiments and generated the FEA simulation data. J.L. developed the surrogate model and design optimization pipeline. All contributed to the drafting and revision of this manuscript.

## Conflicts of Interest

The authors declare no conflicts of interest.

## Conflicts of Interest

The authors declare no conflict of interest.

## Supporting information


**Supporting File 1**: advs73744‐sup‐0001‐movieS1.mp4.

**Supporting File 2**: advs73744‐sup‐0002‐movieS2.mp4.

## Figures and Tables

**FIGURE A1:**
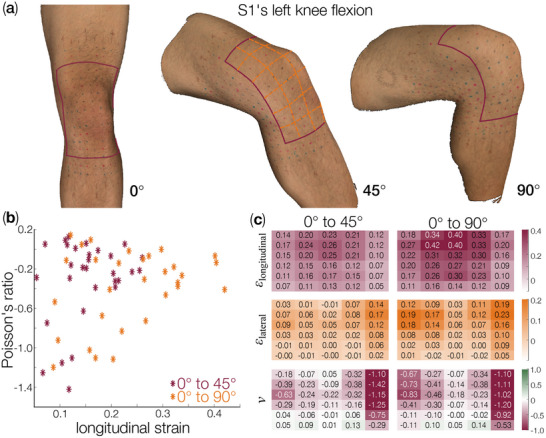
Additional data on subject S1's knee deformation at different joint angles. (a) 3D scan of S1's left knee at 0°, 45°, and 90° flexion angles. (b) The calculated longitudinal strain and Poisson's ratio from the grid cells. One can observe that, as the knee bends from 45° further to 90°, its strain magnitudes increase, but Poisson's ratios remain in a similar range. (c) The heat maps of skin deformation. The three rows are the longitudinal strain, lateral strain, and Poisson's ratio, respectively.

**FIGURE A2:**
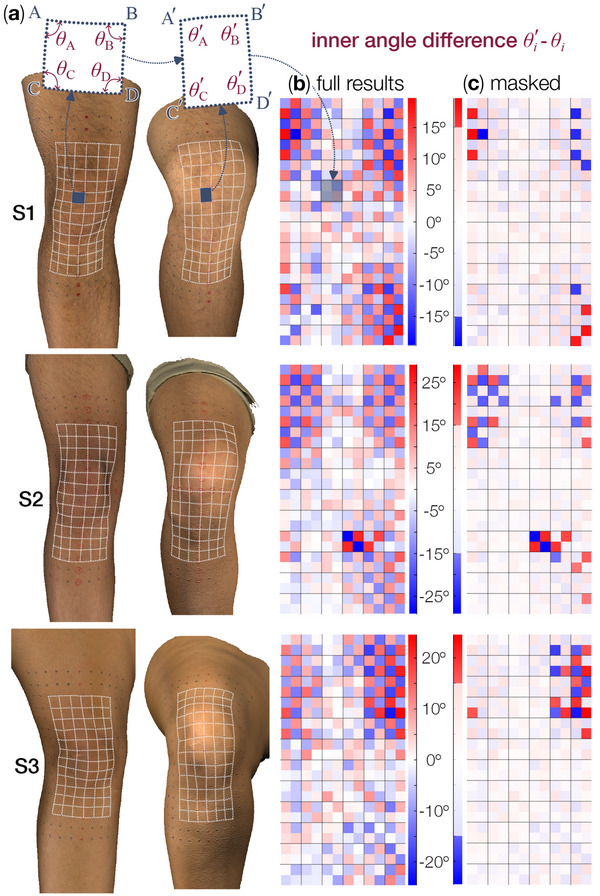
Assessing participant skin's shearing deformation during knee flexion. (a) Another look at the 3D scanning data with the grid cell highlighted. Each grid cell has four internal angles: θ_A_, θ_B_, θ_C_, and θ_D_. We calculate the changes in these internal angles to represent shearing deformation. (b) A heatmap summarizing the results of the angle change calculation. (c) The same heatmap, but the grid cells are masked out if their internal angle changes are smaller than 15°. By examining the two sets of heatmaps, one can see that the shearing deformation are present on the upper knee surface but relatively minor.

**FIGURE A3:**
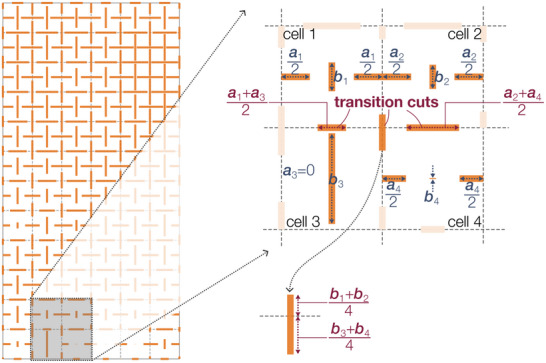
Averaging and skewing of transition cuts between adjacent kirigami unit cells. The lengths of internal cuts within each unit cell are labeled by blue arrows. The lengths of transition cuts are labeled by the red arrows.

## Data Availability

The data that support the findings of this study are available from the corresponding author upon reasonable request.

## Appendix A Knee Skin Deformation at Intermediate Bending Angles

To assess the knee skin deformation between the targets (i.e., between initial and final knee joint angles), we repeated the 3D scanning process on Subject S1's left knee. The anterior surface of the knee was marked with grid points and scanned at 0°, 90°, and an additional intermediate angle of 45° (Figure A1a). Using the GigaMesh software [61], we extracted the geodesic length of the grid cells' edges at different joint angles. This way, we can use the change in these edge lengths to calculate the longitudinal strain, lateral strain, and Poisson's (as described in Section 2.1 of the main text).

Now, comparing the strain distribution at different joint angles (Figure A1b), one can observe that as the subject flexes his knee from 0° to 45°, the Poisson's ratios (maroon color) remain in a similar range to those from the 0‐to‐90° flexion (orange color). The longitudinal strains, on the other hand, increase monotonically from 0°, 45° to 90°. Similar to Figure 2 in the main text, we plot the deformation heatmaps (longitudinal strain, lateral strain, and Poisson's ratio) from 0° to 45°, and 0° to 90°, respectively (Figure A1c). Naturally, the strain magnitudes increase as the joint angle increases; however, the distribution of Poisson's ratio of the intermediate angle is similar to that of a greater angle. That is, both sets of heatmaps show a greater negative Poisson's ratio at the lateral and medial sides of the knee, with a smaller magnitude in the superior region of the patella, and some positive Poisson's ratio in the inferior region.

Therefore, it is reasonable to expect that the kirigami cuts designed for conformability at a greater joint angle (90° in this case) will also reasonably conform to lower intermediate angles. As the knee joint angle dictates the opening of kirigami cuts, the cuts inherently open less at a lower joint angle and more at a greater joint angle — all in an intuitive attempt to conform to the skin surface underneath. These deductions are satisfactorily supported by the close‐up images in Figure 6d of the main text. As the kirigami patch is not designed explicitly according to the initial curvature of the knees, some local slack (wrinkles) are visible when the knee is straight. However, as the knee joint angle increases, this slack is reduced, and the kirigami cuts open in an attempt to conform to the knee surface.

## Appendix B Assessing the Shearing Deformation of Knee Skin

In addition to linear strains, we also computed the shear strain of the knee grid for all three subjects as seen in Figure A2. For this purpose, we first recorded the geodesic length of the two diagonals of the grid cells using the GigaMesh software. Then, we calculate the internal angle at each vertex of the grid cell using the cosine law. For example, the upper left θ_A_ in the example grid cell in Figure A2a can be obtained by

(A1)
$$\theta_{A}=\cos^{-1}\left(\frac{{\hat{AB}}^{2}+{\hat{AC}}^{2}-{\hat{BC}}^{2}}{2\hat{AB}\hat{AC}}\right)$$

We repeated this process for the entire grid, at both 0° and 90° knee joint angles, for all three subjects. After finding the internal angles at each vertex, we calculated their differences and plotted this change in Figure A2b. Such internal angle differences represent the shear strain. The shear strain distributions vary between subjects.

Although these heatmaps show internal angle changes in the range of 0° ± 25° at different regions of the knee, most of the cells show an angular distortion of less than 15°. (To visualize this, we masked out the cells with <15° internal angle changes in Figure A2c). Moreover, cells with shear deformation larger than 15° are mostly positioned at the medial and lateral boundaries of the grid. Even though these shear deformations limit the conformability of our kirigami patch, their impacts are localized. Therefore, to simplify the design process and make our kirigami knee patch rapidly manufacturable, we decided to accept the trade‐off and omit the effect of shear deformation.

## Appendix C Averaging and Skewing Transition Cuts Between Kirigami Unit Cells

The final kirigami patches have heterogeneous designs, each unit cell has a unique cut length *a* and *b*, and these length assignments apply to the cuts *within* the unit cell. However, there are also transition cuts positioned at the boundary *between* unit cells. To this end, we carefully averaged and skewed these transition cuts to ensure a smooth transition.

For example, in Figure A3, we show 4 adjacent unit cells. Between these four cells, there are three transition cuts, two of which are horizontal and one vertical. The horizontal transition cuts between two vertically adjacent cells are simply averaged in length (i.e., (*a*_1_ + *a*_3_)/2, (*a*_2_ + *a*_4_)/2) and centrally placed at the unit cell's boundary. The vertical transition cut, on the other hand, lies at the intersection between all four unit cells. So its length is the average of all four vertical cuts (i.e., (*b*_1_ + *b*_2_ + *b*_3_ + *b*_4_)/4) and its position is skewed accordingly (see details in the zoom‐in view of Figure A3).
